# Association Between FADS1 Gene Polymorphism (rs174549) and Chronic Periodontitis: A Cross-Sectional Study

**DOI:** 10.7759/cureus.63268

**Published:** 2024-06-27

**Authors:** Johnisha Harris, Arvina Rajasekar

**Affiliations:** 1 Periodontics, Saveetha Dental College and Hospitals, Saveetha Institute of Medical and Technical Sciences, Saveetha University, Chennai, IND

**Keywords:** alleles, genotypes, fads1 gene, gene polymorphism, periodontitis

## Abstract

Introduction

FADS1 (fatty acid desaturase 1) gene polymorphism results in more susceptibility to certain metabolic diseases and chronic inflammatory diseases like periodontitis. This study aims to analyze the association between FADS1 gene polymorphism and various stages of periodontitis.

Materials and methods

One hundred subjects included in the study were categorized into two groups: group A (n = 50) had healthy periodontium, and group B (n = 50) had ≥stage II periodontitis. They were graded based on the clinical parameters of probing pocket depth (PPD), clinical attachment level (CAL), and bleeding on probing (BOP). Five milliliters of venous blood were collected, and DNA isolation was done. Genomic DNA was extracted. The DNA was then subjected to amplification with the help of specific primers flanking the *Providencia stuartii* I (PstI) polymorphic site of the FADS1 gene. A chi-square test aimed to examine the genotype and allele frequency distributions in both groups; p < 0.05 was considered statistically significant.

Results

The difference in genotype frequency of FADS1 polymorphism was statistically insignificant (p = 0.91). Our study revealed no significant difference (AA vs. AG+GG) between the periodontitis and control groups between homozygous and heterozygous variant genotypes with a p-value of 0.7764. The frequency of AG (28% vs. 30%) and GG (62% vs. 58%) genotypes showed no significant difference between the periodontitis group and healthy control subjects. No significant difference was seen in the G allele (77% vs. 73%) and A allele (23% vs. 27%) between the periodontitis and control groups.

Conclusion

The study concluded that FADS1 receptor polymorphism is not associated with periodontitis in the study population.

## Introduction

Periodontitis is an inflammatory disease of the periodontium primarily caused by gram-negative anaerobic bacteria, but the spread of the disease is also influenced by environmental and genetic variables [1]. The host’s susceptibility to periodontitis is mainly influenced by the individual’s genetics. The search to find the genetic factors responsible for periodontal disease is ongoing. Various studies have been conducted to identify the genetic factors responsible for this.

Single nucleotide polymorphism (SNP) is a genetic variation that occurs when a single nucleotide at a specific position in the DNA sequence differs among individuals. SNPs are the most common genetic variation in the human genome and are responsible for much of the genetic diversity between individuals [2]. SNPs in the fatty acid desaturase (FADS) gene cluster (FADS 1-2-3) have been identified frequently across cardiovascular diseases, obesity, and diabetes mellitus. However, the relationship linking the FADS 1-2-3 gene polymorphism and various grades of periodontitis is not given much attention [3].

The FADS1 gene, also known as fatty acid desaturase 1, is located on chromosome 11q12.2. It encodes an enzyme called delta-5 desaturase, which is involved in the conversion of certain fatty acids in the body. The delta-5 desaturase enzyme catalyzes the change from dihomo-gamma-linolenic acid (DGLA) to arachidonic acid (AA), an essential omega-6 polyunsaturated fatty acid [4]. Genetic variations in the FADS1 gene have been extensively studied, particularly SNPs, which can affect the enzyme’s activity and subsequent fatty acid metabolism [5]. One of the most well-known SNPs in the FADS1 gene is rs174549. The amount of total cholesterol and high-density lipoprotein are influenced by FADS1 and FADS2 genes, which leads to more susceptible metabolic and chronic inflammatory diseases like periodontitis [6]. Although some literature has mentioned it, the relationship between FADS1 gene polymorphism and periodontitis has not been extensively explored. This study aimed to examine the association between FADS1 gene polymorphism and various stages of periodontitis.

## Materials and methods

In this cross-sectional study, 100 outpatient subjects were recruited from the Department of Periodontics at Saveetha Dental College, Chennai. The sample size was calculated using G*power. The study population was divided into two groups based on the following clinical parameters: probing pocket depth (PPD), clinical attachment loss (CAL), and bleeding on probing (BOP). Group A (n = 50) was the control group with healthy periodontium and a mean age of 41.34 ± 7.49 years. Group B (n = 50) was the test group with stage II periodontitis and above, with a mean age of 39.02 ± 8.22 years. They were graded according to the clinical parameters of PPD, CAL, and BOP. The periodontitis patients were recruited based on the criteria of the American Academy of Periodontology 2018.

A detailed history regarding general health conditions, history of dental treatment, familial history of periodontitis, and other habits were obtained from the subjects. Pregnant and lactating women, patients with smoking habits, systemically compromised patients, those under medication, and patients who had undergone periodontal therapy in the past six months were excluded from the study [7]. The study was done after receiving approval from the Institutional Human Ethical Committee (IHEC/SDC/PERIO-2103/23/075).

Sample collection and DNA extraction

Five milliliters of venous blood were collected, dispersed in a sterile tube containing ethylenediaminetetraacetic acid (EDTA), and mixed well to prevent clotting of the collected blood. DNA isolation was done in accordance with the modified Miller et al. 1998 protocol [8].

Polymerase chain reaction and restriction endonuclease digestion

**Figure 1:**
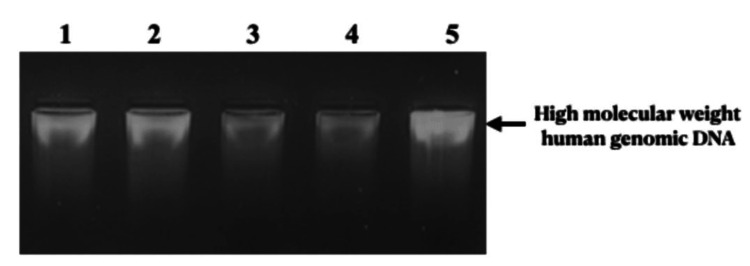
High molecular weight human genomic DNA isolated from peripheral blood samples.

**Figure 2:**
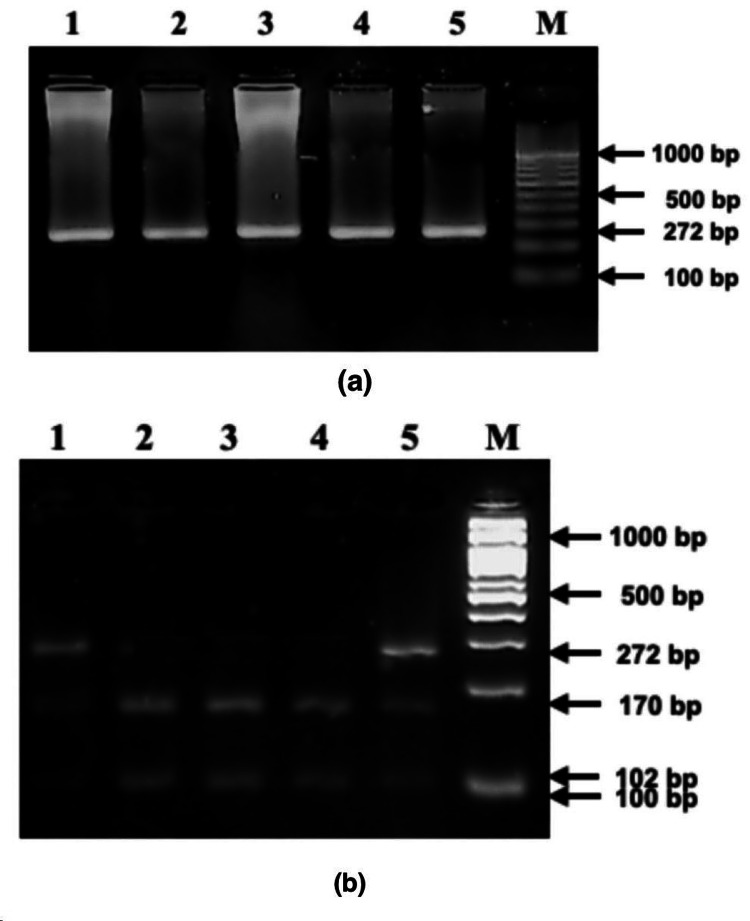
(a) Agarose gel electrophoretogram of FADS1 (rs174549) showing 272 bp amplicon in lanes 2-6 (lane 7 (M): 100 bp DNA ladder). Lane 1: negative control. (b) PstI digestion of PCR amplified product (lanes 1 - AA wild type; lanes 2-4 - GG-homozygous variant; lane 5-AG - heterozygous; lane 6 - 100 bp DNA ladder).

By using restriction digestion and polymerase chain reaction (PCR) amplification, the polymorphisms of the FADS1 receptor gene were evaluated. Using the forward primer 5′‐CTCTTCCTCTCCTC CAGCAG‐3′ and the reverse primer 5′‐GAGCCATGGTCCTGGCAGATT‐3′, DNA spanning the FADS1 gene’s Providencia stuartii I (PstI) polymorphism site was amplified. DNA amplification was carried out in 20 μL of PCR Master Mix (Takara, Shiga, Japan), 5 pmol/μL forward and reverse primers, and 10 ng of genomic DNA. The cycle parameters were as follows: five minutes of initial denaturation at 94°C, 35 seconds of denaturation at 94°C, 35 seconds of annealing at 60°C, 35 seconds of extension at 72°C, and five minutes of final extension at 72°C. Next, 15 μL of the PCR product was digested with a PstI restriction enzyme (New England Biolabs, Hitchin, UK), after which a 5 μL portion of the product was examined on a 1% agarose gel. The digestion process lasted two hours at 37°C [8]. The digested product was seen on a 2% agarose gel, and the outcomes were recorded as shown in Figures 1-2.

Primers

Table 1 shows the forward and reverse primers used for amplification of DNA spanning the PstI polymorphic site of the FADS1 gene.

**Table 1:** Forward and reverse primers used for amplification of DNA spanning the PstI polymorphic site of the FADS1 gene RFLP enzyme used: PstI - type II restriction endonuclease PstI - *Providencia stuartii* I

Amplicon size	Primer name	Sequence
272 bp	FADS1-F	5'-CTCTT CCTC TCCTC CAGCAG-3’
	FADS1-R	5’-GAGCCATGGTCCTGGCAGATT-3’

Statistical analysis

Statistical analysis was done using SPSS version 23.0, which revealed that the frequency of the genotype of FADS1 polymorphism showed no statistically significant difference (p = 0.91). With a p-value of 0.7764, our study’s findings demonstrated no significant difference in the prevalence of homozygous and heterozygous variant genotypes (AA vs. AG+GG) between the two groups. There was no difference in the detected frequency of the AG (28% vs. 30%) and GG (62% vs. 58%) genotypes between the participants with periodontitis and the healthy control group. The G allele (77% vs. 73%) and the A allele (23% vs. 27%) did not significantly differ between the groups.

## Results

Table 2 shows the clinical parameters of the subjects in the control group and periodontitis groups.

**Table 2:** Demographic details CAL - clinical attachment loss; GI - gingival index; PPD - probing pocket depth

SN	Groups	No. of subjects			Mean age	CAL	PPD	GI
		Male	Female	Total				
1	Periodontitis	26	24	50	39.02 ± 8.22	6.13±1.29	5.48 ± 1.15	1.74 ± 0.22
2	Control	26	24	50	41.34 ± 7.488	-	1.60 ± 0.57	0.76 ± 0.16

Tables 3-4 show the group distributions and genotype frequencies. There was no statistically significant difference in the genotype frequency of FADS1 polymorphism (p = 0.91). With a p-value of 0.7764, our study's findings demonstrated that there was no significant difference in the prevalence of homozygous and heterozygous variant genotypes (AA vs. AG+GG) between the periodontitis and control groups (Table 3).

**Table 3:** Genotype frequencies of FADS1 (rs174549) in both groups FADS1 - fatty acid desaturase 1; AA, GG - homozygous allele; AG - heterozygous allele

Groups (n = 50)	AA	AG	GG	G	A	p Value*
Case	5	14	31	0.77	0.23	0.100
Control	6	15	29	0.73	0.27	0.091

There was no discernible difference in the detected frequency of the AG (28% vs. 30%) and GG (62% vs. 58%) genotypes between the participants with periodontitis and the healthy control group. The G allele (77% vs. 73%) and the A allele (23% vs. 27%) did not significantly differ between the group with periodontitis and the healthy control group (Table 4).

**Table 4:** Overall genotype distribution of the FADS1 (rs174549) gene polymorphism FADS1 - fatty acid desaturase 1

Genotype	Case	Control	Unadjusted OR (95% CI)	p Value
Dominant				
AA	5	6	0.8148	0.7496
AG+GG	45	44	(0.2317-2.86530)	
Recessive				
AG+AA	19	21	0.8464	0.6832
GG	31	29	(0.3799-1.8855)	
Allele				
A	23	27	0.8076	0.5140
G	77	73	(0.4516-1.5343)	

## Discussion

Periodontitis is primarily caused by bacterial plaque but can also be influenced by genetic and environmental factors [9]. Genetic polymorphism can influence illness susceptibility and progression in a multitude of intricate ways by interacting with other genetic variants and environmental factors [10]. Genetic variants in the FADS gene cluster have recently been linked to the deposition of substrates and decreased desaturase reaction products, which may have consequences for cardiovascular disease [11]. SNPs in the FADS1 and FADS2 group of genes have been known to be strongly linked to an increased risk of cardiovascular disease. Association between FADS1 and FADS2 variations and the concentration of lipid levels in the plasma have also been identified in other investigations. Literature research has established that the FADS gene variation is linked to numerous chronic illnesses [3]. However, its correlation with periodontitis has not been investigated.

Studies investigating the connection between FADS1 and susceptibility to periodontitis are scarce. Our investigation revealed that the frequency of the genotype in FADS1 polymorphism did not change significantly (p = 0.937). There was no significant difference between the chronic periodontitis and control groups (AA vs. AG + GG) in the frequency of both homozygous and heterozygous genotypes (p = 0.7764). The frequency of G and A genotypes (PRP2) showed no significant statistical difference between the CP and control groups, respectively. When compared to the periodontally healthy control group, the GG genotype and G allele were found to be expressed at a higher frequency in the periodontitis population. This suggests that the presence of genotype GG may be a risk indicator for periodontitis. The study also showed that the detection rate of genotype GG in the group with periodontitis was greater than in the group with healthy periodontium.

FADS1 rs174537 polymorphism has been linked to aggressive periodontitis, according to the study by Song et al. [12]. In group AgP, the patients carrying the GG genotype had comparatively reduce total protein (TP), globulin (GLB), and higher albumin/globulin (A/G). Because genotype GG lowers host defense capacity and promotes an inflammatory response in the occurrence and development of aggressive periodontitis, it may be an indicator of the disease.

Henry et al. discovered that patients with severe periodontitis had a considerably higher frequency of TNF−σ+308 allele 1 than those with gingivitis associated with plaque [13]. The severity of periodontal destruction was reported to be associated with periodontitis by Galbraith et al. [14] and with the R-allele by Grobe et al. [15] and Lopez et al. [16]. According to Moreira et al. [17] and Wagner et al. [18], chronic periodontitis is linked to the IL1B +3954 R-allele. Nevertheless, in chronic periodontitis, Rogers et al. found an association with the N-allele rather than the R-allele [19]. According to a study conducted among South Indians, Murthykumar et al. found no significant connection between the VDR gene FokI (rs10735810) polymorphism and chronic periodontitis [20]. Another study by Karthikeyan et al. revealed that TaqI DR gene polymorphism is linked to chronic periodontitis and that the Tt and tt genotypes had a greater probability of occurring in chronic periodontitis [21]. A distinct allele of an SNP may be a genetic risk factor for disease susceptibility in one community but not in another, according to Heidari et al. [22]. Görgün et al. discovered no correlation between the IL-13 -1112 C/T and -1512 A/C gene polymorphisms and generalized aggressive periodontitis [23]. Karthikeyan et al. found that the MMP8 (-799C/T) (rs11225395) polymorphism had no significant connection with chronic periodontitis [24]. However, the FADS1 gene and chronic periodontitis did not significantly correlate in the current study.

Limitations

Small research sample and homogeneous subject population are the main limitations of the study. Therefore, future research should concentrate on determining the expression of the FADS1 gene in the gingiva to gather additional evidence supporting the hypothesis that any genetic variation or mutation in the FADS1 receptors is linked to periodontitis. Periodontal disease treatment strategies can be based on this, which can provide compelling evidence of a link between periodontitis and the FADS1 gene polymorphism.

## Conclusions

The study concluded that FADS1 receptor polymorphism is not associated with periodontitis stage II and above in the study group analyzed. More research is needed to explore the interaction of the FADS1 receptor gene with microbial and environmental factors in the pathogenesis of periodontitis, as well as the association between the FADS1 receptor gene and systemic diseases in periodontitis patients. Future research endeavors ought to concentrate on associating the polymorphism of the FADS1 receptor gene with periodontitis and other systemic diseases such as diabetes mellitus and cardiovascular disease.
